# Pharmacological Inactivation of Src Family Kinases Inhibits LPS-Induced TNF-*α* Production in PBMC of Patients with Behçet's Disease

**DOI:** 10.1155/2016/5414369

**Published:** 2016-06-30

**Authors:** Sevgi Irtegun, Gulsum Pektanc, Zeynep M. Akkurt, Mehtap Bozkurt, Fatih M. Turkcu, Sevgi Kalkanli-Tas

**Affiliations:** ^1^Department of Medical Biology, Faculty of Medicine, Dicle University, 21280 Diyarbakır, Turkey; ^2^Department of Dermatology, Faculty of Medicine, Dicle University, 21280 Diyarbakır, Turkey; ^3^Department of Physical Therapy and Rehabilitation, Faculty of Medicine, Dicle University, 21280 Diyarbakır, Turkey; ^4^Department of Ophthalmology, Faculty of Medicine, Dicle University, 21280 Diyarbakır, Turkey; ^5^Department of Immunology, Faculty of Medicine, Dicle University, 21280 Diyarbakır, Turkey

## Abstract

Behçet's disease (BD) is a multisystemic chronic inflammatory disease characterized by relapsing oral and genital ulcers, uveitis, and skin lesions. The pathogenesis of BD is still unknown. Aberrant production of some cytokines/chemokines plays an important role in the pathogenesis of various inflammatory diseases. Revealing a key signaling regulatory mechanism involved in proinflammatory cytokines/chemokines production is critical for understanding of the pathogenesis of BD. The aim of this study was to determine the role of Src family kinases (SFKs) in production of some LPS-induced proinflammatory cytokines/chemokines in peripheral blood mononuclear cells (PBMC) of active BD patients. Chemical inhibition of SFKs activity impaired LPS-induced TNF-*α* production in PBMC of active BD patients, suggesting that modulating SFKs activity may be a potential target for BD treatment.

## 1. Introduction

Behçet's disease (BD) is a chronic inflammatory disorder characterized by recurrent oral and genital ulcers, uveitis, and skin lesions [1, 2]. BD is a multisystemic disease due to involvement of central nervous and gastrointestinal systems, large vessels, joints, heart, and lungs [3]. Although the pathogenesis of the disease remains poorly understood, it is currently believed that immunological abnormalities triggered by some infectious and environmental factors in genetically susceptible individuals may play crucial roles during the pathogenesis of BD [2]. Indeed, an increasing amount of data has reported that immunological abnormalities are implicated in the BD pathophysiology [4–6].

Cytokines that mediate inflammatory and immune response play an important role in the pathogenesis of BD [2]. Elevated inflammatory response in BD appears to result from increased production of proinflammatory cytokines. Several lines of evidence supported the involvement of proinflammatory cytokines and chemokines in the disease pathogenicity and/or activity [4, 5]. The secretion of IL-6, IL-8, and TNF-*α* has been found to be increased in the serum of BD patients [7–9]. A study has reported that increased serum level of TNF-*α* is associated with active uveitis in BD patients [6]. Furthermore, a study has demonstrated that serum level of IL-8 which is a major chemokine involved in inflammation may be used as a serological marker for BD activity [10]. Similarly, a recent study has also demonstrated that serum level of IL-8 is elevated in active BD patients compared to inactive BD patients, suggesting that increased IL-8 level is associated with disease activity [11]. In another study, expression of IL-6 gene was found to be increased in active BD patients and IL-6 level was shown to increase in supernatant of cultured PBMC isolated from active BD patients [12].

Lipopolysaccharide (LPS) is a major component of the outer cell wall of Gram-negative bacteria causing acute inflammation. LPS binds to toll-like receptor-4 (TLR4) to exert its inflammatory action [13]. Ligation of TLR4 by LPS triggers activation of signaling cascades involved in inflammatory response [14, 15]. LPS stimulates the activation of immune system cells, including monocytes and macrophages [16, 17]. In cell culture, LPS elicits inflammatory response by rapidly inducing production of proinflammatory cytokines and chemokines, including TNF-*α*, IL-6, and IL-8 [12, 18]. Studies reported that Src family kinases (SFKs) are activated upon LPS stimulation and involved in LPS-induced TLR4 signaling pathway [14, 19]. It was reported that LPS-induced TNF-*α* production in monocytes is regulated by SFKs activity. Chemical inhibition of these kinases impaired TNF-*α* secretion in response to LPS [19]. Although it is known that SFKs are implicated in signaling pathways induced by LPS, the role of SFKs in LPS-induced cytokines/chemokines production has yet to be investigated in patients with BD.

In the present study, we have investigated whether SFKs are involved in LPS-induced TNF-*α*, IL-6, and IL-8 increase in PBMC of active BD patients. Age and sex matched healthy subjects were used as controls in the study.

## 2. Materials and Methods

### 2.1. Patients and Control Subjects

Twenty active BD patients (eleven male and nine female) were included in this study. The mean age of active BD patients was 31 years (range 20–41 years shown in Table 1). All patients fulfilled the criteria of the International Study Group for Behçet's Disease [20]. All active BD patients were showing at least three of the major symptoms, including genital ulcers, oral ulcers, skin lesions, and uveitis (Table 1). When blood samples were removed from the BD patients during the active stage of disease, none of the patients was receiving any treatments. Twenty sex and age matched healthy subjects, eleven male and nine female, with a mean age of 31 years (range 20–41 years), were included as controls in the study. The study was approved by the local ethics committee, and written informed consents were taken from all patients and healthy subjects.

### 2.2. Reagents

RPMI 1640 medium, fetal bovine serum (FBS), L-glutamine, and penicillin/streptomycin were from Gibco by Life Technologies, UK. Ficoll-Paque Plus was from GE Healthcare, Sweden. Phosphate-buffered saline (PBS) was from HyClone, Logan, Utah. Dimethyl sulfoxide (DMSO) was from Santa Cruz Biotechnology, USA. PP2 (4-amino-5-(4-chlorophenyl)-7-(dimethylethyl)pyrazolo[3,4-*d*] pyrimidine) was from Sigma Aldrich, USA. Lipopolysaccharides (LPS) from* Escherichia coli* 0111:B4 were from Sigma Aldrich, USA. BD vacutainer heparinized tubes were from BD Biosciences, UK. Enhanced chemiluminescence (ECL) was obtained from Bio-Rad, USA.

### 2.3. Antibodies

Anti-*β*-actin and anti-TNF-*α* antibodies were from Abcam. Anti-Hck and Anti-Yes antibodies were from Santa Cruz Biotechnology. Anti-Lck, anti-Lyn, anti-Fyn, anti-c-Src, and anti-pTyr-416-SFKs antibodies were from Cell Signaling Technology. HRP-conjugated goat anti-mouse and goat anti-rabbit antibodies were from Abcam.

### 2.4. Cell Separation and Culture

Peripheral blood mononuclear cells (PBMC) were separated from 15 mL heparinized venous blood that was mixed with 15 mL PBS, using Ficoll-Paque Plus density gradient centrifugation [21]. The cells were washed twice in PBS and then resuspended in RPMI 1640 medium supplemented with 10% fetal bovine serum (FBS), 2 mM L-glutamine, and 100 units/mL penicillin/streptomycin (all from Life Technologies). The former cell suspensions were finally adjusted to a concentration of 2 × 10^6^ cells/mL. All cells culture incubations were performed at 37°C in a humidified atmosphere containing 5% CO_2_.

### 2.5. ELISA

PBMC were plated at 5 × 10^5^ cells/well on a 96-well plate. To suppress the SFKs activity, PBMC were treated with 10 *μ*M PP2, a selective and potent SFKs inhibitor, for 1 h before 100 ng/mL LPS stimulation for 18 h. The cells were treated with DMSO to be used as control. Supernatants were harvested after 18 h and examined for concentrations of TNF-*α*, IL-6, and IL-8 by ELISA (BOSTER Immunoleader, USA) following the manufacturers' instructions. Absorbance was read at 450 nm on a spectrophotometric plate reader (Multiskan GO, Thermo Scientific) and analyzed using the SkanIt software program. The limits of detection for individual cytokines and chemokine were as follows: TNF-*α*, 15.6 pg/mL; IL-6, 4.69 pg/mL; IL-8, 15.6 pg/mL.

### 2.6. Western Blot Analysis

PBMC were plated at 5 × 10^6^ cells/well on a 6-well plate. To inhibit the SFKs activity, PBMC were treated with 10 *μ*M PP2 for 1 h before 100 ng/mL LPS stimulation for 15 min. The cells were treated with DMSO to be used as control. After treatments, the cells were washed with ice-cold PBS and lysed on ice in RIPA buffer (Sigma Aldrich) supplemented with protease and phosphatase inhibitor cocktail (Thermo Scientific). Total cellular protein concentration was determined using a BCA protein assay kit according to the manufacturer's instructions (Pierce, Thermo Scientific). Total cellular proteins (20 *μ*g) were separated by 10% SDS-PAGE gel and separated proteins from the SDS-PAGE were transferred onto polyvinyl difluoride (PVDF) membrane (Bio-Rad). Nonspecific binding was blocked by membrane incubation in PBS with 5% nonfat dried milk and 0.05% Tween-20 for 1 h at room temperature. The membranes were probed with primary antibodies for 2 h at room temperature. The total protein levels of Src family members (Hck, Lyn, Fyn, c-Src, Lck, and Yes) and TNF-*α* were detected with antibodies against the indicated proteins. *β*-actin was used as a loading control. Phosphorylation levels of SFKs were detected using anti-pTyr-416-SFKs antibody. In the literature, phosphorylation level of Tyr-416 has been extensively used as a critical determinant of SFKs catalytic activity [22–26]. Appropriate HRP-conjugated secondary antibodies were used to visualize the specific bands. The protein bands were visualized using ECL (Bio-Rad) according to the manufacturer's instruction. The images were taken using ChemiDoc*™* MP (Bio-Rad).

### 2.7. Statistical Analysis

Significance of the results has been evaluated by Student's *t*-test using the SigmaPlot 12 software package (Systat). A *p* value of < 0.05 was considered statistically significant.

## 3. Results

**Figure 1:**
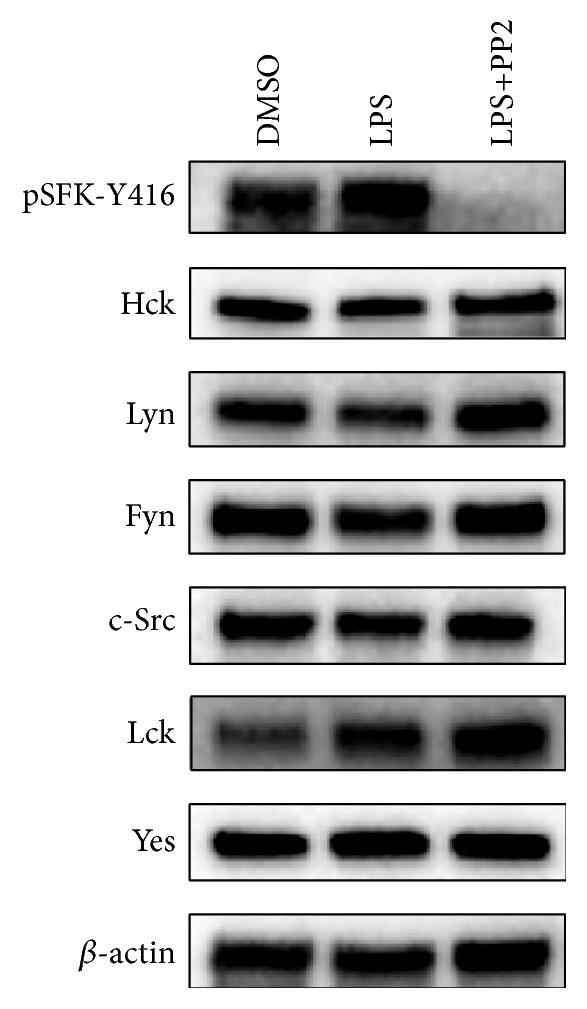
Expression of SFKs in PBMC of active BD patients. PBMC were isolated from total blood and 5 × 10^6^ PBMC were cultured. The cells were treated with 10 *μ*M PP2 for 1 h before 100 ng/mL LPS stimulation for 15 min. DMSO treated cells were used as control. Cell lysates were examined for the presence of SFKs and activity of SFKs by Western Blotting. The lowest panel represents loading control (*β*-actin). The image shown represents a single representative example of five separate experiments.

It was found that Hck, Lyn, Fyn, c-Src, Lck, and Yes were expressed abundantly in PBMC of active BD patients (Figure 1). However, there was no detectable expression of Blk and Fgr, which are other Src family members (data not shown). While LPS stimulation led to an increase in SFKs activity, SFKs activity was abolished by PP2 treatment in PBMC of active BD patients (Figure 1).

**Figure 2:**
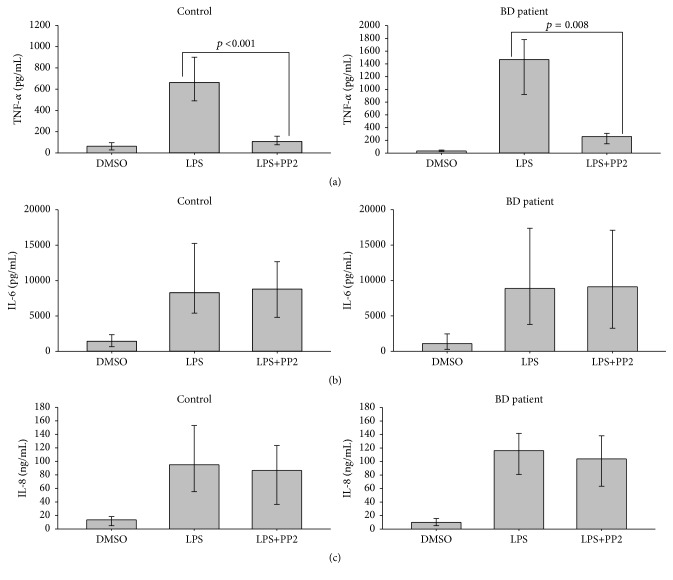
The effect of the PP2 on LPS-induced cytokine/chemokine production in PBMC. 0.5 × 10^6^ PBMC were treated with 10 *μ*M PP2 for 1 h prior to stimulation with 100 ng/mL LPS for 18 h. Supernatants were collected and cytokine/chemokine levels determined by ELISA; TNF-*α* (a), IL-6 (b), and IL-8 (c). The results are presented as pooled data from three independent experiments. The statistical significance of the data was analyzed by Student's *t*-test.

ELISA results showed that suppression of SFKs activity by PP2 blocked LPS-induced TNF-*α* increase in the PBMC of both controls and active BD patients (Figure 2(a)). However, the inhibition of SFKs activity had no significant effect on LPS-induced IL-6 and IL-8 release (Figures 2(b) and 2(c)).

**Figure 3:**
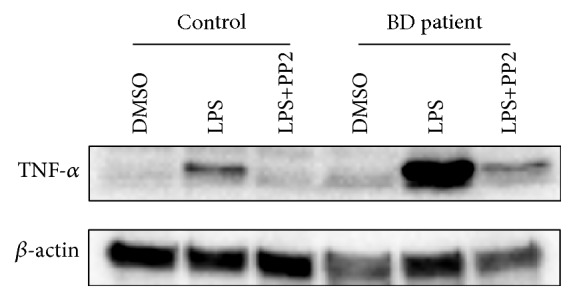
Inhibition of LPS-induced TNF-*α* production by PP2 in PBMC of active BD patients. Five × 10^6^ PBMC were treated with 10 *μ*M PP2 for 1 h before 100 ng/mL LPS stimulation for 18 h. Cell lysates were examined for the presence of TNF-*α* by Western Blotting. The lowest panel represents loading control (*β*-actin). The image shown represents a single representative example of four separate experiments.

Similar results were obtained with Western Blotting. The results showed that LPS stimulation led to a dramatic increase in TNF-*α* protein level of PBMC of active BD patients compared to PBMC of control. However, suppressing the SFKs activity by PP2 inhibited LPS-induced TNF-*α* increase (Figure 3). As a result, inhibition of SFKs activity in PBMC of active BD patients impaired TNF-*α* production upon LPS treatment.

## 4. Discussion

In this study, we demonstrated for the first time the functional significance of SFKs activity in the LPS-induced TNF-*α* production in PBMC of active BD patients. Studies have reported that SFKs are involved in inflammatory diseases, including rheumatoid arthritis and osteoarthritis [27, 28], but the role of SFKs in the pathogenesis of BD has not been investigated yet. To our knowledge, there is no report available on the role of SFKs in LPS-induced cytokines/chemokines production in PBMC of active BD patients.

Here we showed that six SFK members (Hck, Lyn, Fyn, c-Src, Lck, and Yes) are expressed in PBMC, but Fgr and Blk are not expressed at detectable levels (data not shown). We found that LPS stimulation led to an increase in SFKs activity. This result is in good agreement with previous studies reporting that LPS induces activation of SFKs [17–19]. The role of SFKs in LPS-induced production of TNF-*α* in monocytes and macrophages is reported in literature [19, 29, 30]. However, a definitive evidence for a role of SFKs in PBMC of BD patients remains unknown. It is known that PBMC can be stimulated by LPS to produce high amounts of cytokines/chemokines, such as TNF-*α*, IL-6, and IL-8 [19, 31]. Toll-like receptors (TLR) play an important role in pathogen recognition and activation of innate immunity. Engagement of TLR4 by LPS induces activation of signaling pathways leading to increased inflammatory cytokines/chemokines production [14, 15, 31, 32]. Some evidence showed that SFKs are involved in signaling pathways triggered by LPS [17, 18, 32].

We observed that PBMC of active BD patients showed a significant response compared to healthy subjects upon LPS stimulation. Stimulation of PBMC with LPS resulted in a significant increase in TNF-*α* production, but this increase in PBMC of BD patients was much higher compared to healthy subjects used as controls. We found that inhibition of SFKs activity by PP2 dramatically blocked LPS-induced TNF-*α* release. TNF-*α* is the most important cytokine in the initiation and progression of inflammatory disease. As TNF-*α* is involved in the pathogenesis of chronic inflammatory conditions, an increased TNF-*α* level is associated with destructive inflammatory process in many inflammatory diseases, including BD [33–35]. Indeed, previous studies reported increased serum level of TNF-*α* in active BD patients [36, 37]. Our results suggest that SFKs activity is required for LPS-induced TNF-*α* production in PBMC of active BD patients.

It is known that LPS-induced TLR4 signaling leads to an increase in TNF-*α* protein level [38]. Here we also showed that TNF-*α* protein level was increased in response to LPS stimulation. Interestingly, PBMC of BD patients gave a much higher response to the LPS stimulation than PBMC of controls. The amount of TNF-*α* protein level in PBMC of BD patients was significantly increased compared to PBMC of healthy subjects upon LPS stimulation. LPS-induced TNF-*α* production was suppressed by chemical inhibition of SFKs activity. This result indicates that PBMC of BD patients is more prone to production of inflammatory TNF-*α* cytokine associated with inflammatory disorders and SFKs activity is necessary for LPS-induced TNF-*α* production in PBMC of active BD patients. To our knowledge, this is the first example showing the pharmacological inhibition of SFKs activity is able to modulate LPS-induced TNF-*α* production in PBMC of active BD patients.

Herein, we showed that IL-6 and IL-8 levels in PBMC of BD patients were also increased in response to LPS stimulation. A previous study reported that LPS-induced IL-6 production was reduced by 50% upon PP2 treatment in primary human macrophages [19]. However, our results revealed that LPS-induced IL-6 and IL-8 production were not significantly affected by the chemical inhibition of SFKs activity. One possible explanation for this result is that SFKs have a differential effect on LPS-induced inflammatory cytokines production in PBMC. SFKs might be a potential future drug target for BD treatment. Further study is required to elucidate which SFKs members are exactly required for regulation of TLR4 involved signaling pathways in BD.

## 5. Conclusion

In conclusion, this study has investigated the role of SFKs on LPS-induced inflammatory cytokines/chemokines production in PBMC of active BD patients. Blocking SFKs activity by PP2 resulted in inhibition of LPS-induced TNF-*α* production, suggesting that SFKs are a key component of TLR4 involved signaling pathways. Further study is required to elucidate which SFKs members are exactly required for regulation of TLR4 involved signaling pathways in BD. Taken together, SFKs might be a potential future drug target for BD treatment.

## Figures and Tables

**Table 1:** Demographic and clinical features of active BD patients.

Patients (*n*: 20)	Age (years)	Sex (F/M)	Oral ulcer	Genital ulcer	Arthritis	Skin lesions	Uveitis	Positive pathergy test
1	33	F	+	−	+	+	+	+
2	25	M	+	+	+	+	−	−
3	33	F	−	+	+	+	+	+
4	32	M	+	−	+	+	−	−
5	31	M	+	−	+	+	+	+
6	36	M	+	+	+	−	+	−
7	34	M	+	−	+	−	+	−
8	31	M	+	−	+	+	+	+
9	28	F	+	+	−	+	−	−
10	27	F	+	−	−	+	+	+
11	26	F	+	+	−	+	−	+
12	20	F	+	+	−	+	−	+
13	39	M	+	+	+	+	−	+
14	33	F	+	+	+	−	+	+
15	25	F	+	+	+	+	−	−
16	27	F	+	+	−	+	−	+
17	41	M	+	+	−	+	+	+
18	37	M	+	+	+	+	−	−
19	28	M	+	+	−	+	−	+
20	34	M	+	+	−	−	+	+

F: female; M: male.
